# The Infallible Microscope

**Published:** 1884-07

**Authors:** A. McNeil


### "THE INFALLIBLE MICROSCOPE."

The comments on page 115 of your journal as to mistaken diagnoses, based on the so-called cancer cells, leads me to translate a few lines from Hebra's classical work. It does seem strange that American practitioners, even of eminence, should speak of the microscope as the only test of the malignant character of a growth. In *Hebra und Kaposi's Hautkrankheiten*, Band II, page 436, is the following :

"Some pathologists believed that they discovered constituents of the tumor, which they designated cancer cells. But, however, a closer investigation of the subject has demonstrated that these constituents of cancer cannot be distinguished from the equivocal (*alquivoquom*) physiological structures (*epithelia*) within by their morphological properties, nor by their striking *endogenous* proliferation, nor by their occasional pigment. (Virchow, *Zur Entwickelungsgeschichte des Krebses*, etc. *Dessen Archio*, B. I, 104.) Therefore, the idea of the existence of specific cancer cells must be abandoned as unproven or unprovable."

Billroth's views agree with Virchow. These names are weighty enough to settle this question till newer light and stronger demonstrations overthrow them.

A. McNeil.

